# When a Peripartum Cardiomyopathy Patient Hides Various and Serious Risk Factors for Recurrent and Fatal Thromboembolic Events Even Under Well-Conducted Oral Anticoagulation

**DOI:** 10.7759/cureus.12392

**Published:** 2020-12-31

**Authors:** Chaimae Toutai, Oussama Kallel, Laachach Houssam, Nabila Ismaili, Noha Elouafi

**Affiliations:** 1 Cardiology, Mohammed I University/Mohammed VI University Hospital/Epidemiological Laboratory of Clinical Research and Public Health, Oujda, MAR; 2 Cardiology, Mohammed I University/Mohammed VI University Hospital, Oujda, MAR

**Keywords:** peripartum dilated cardiomyopathy, thromboembolic events, recurrence, protein c and protein s deficiencies

## Abstract

Peripartum cardiomyopathy (PPCM) is a rare cause of heart failure that occurs during the final month of pregnancy through about five months after delivery, without any other known cause, and it increases the risk of thromboembolic events by many folds. A 38-year-old female with a history of peripartum dilated cardiomyopathy was admitted to our hospital, one month after a cesarean section, for severe breathlessness. Examination revealed signs of global heart failure and right deep vein thrombosis. Pulmonary CT angiography revealed pulmonary embolism of the right pulmonary artery. The patient was treated by oral anticoagulation with acenocoumarol with all international normalized ratio (INR) values within the target range (2-3). One month later, she was admitted to the emergency department with acute dyspnea and superior vena cava syndrome. Thoracic CT angiogram showed bilateral pulmonary emboli associated with an extensive deep vein thrombosis of both internal jugular veins, sigmoid sinuses, subclavian veins, innominate venous trunks, and the origin of the superior vena cava without any lesion suspected of malignancy. The thrombophilia screen performed six weeks after the suspension of vitamin K antagonists (VKAs) revealed severe deficiencies of protein C and protein S. In this report, we present the first case of recurrence of fatal thromboembolic events under well-conducted oral anticoagulation in a patient with PPCM associated with severe protein C and protein S deficiencies.

## Introduction

The risk of thromboembolic events during pregnancy and postpartum is five-fold higher when compared to the general population [1]. Peripartum dilated cardiomyopathy is a rare but life-threatening disease, which, in addition to all the risk factors present during this period, significantly increases the risk of thromboembolic events, which are major causes of long-lasting maternal morbidity and mortality. In this report, we discuss the case of a 38-year-old female in whom the diagnosis of peripartum dilated cardiomyopathy was retained, with a course marked by recurrent thromboembolic events even under well-conducted oral anticoagulation.

## Case presentation

**Figure 1:**
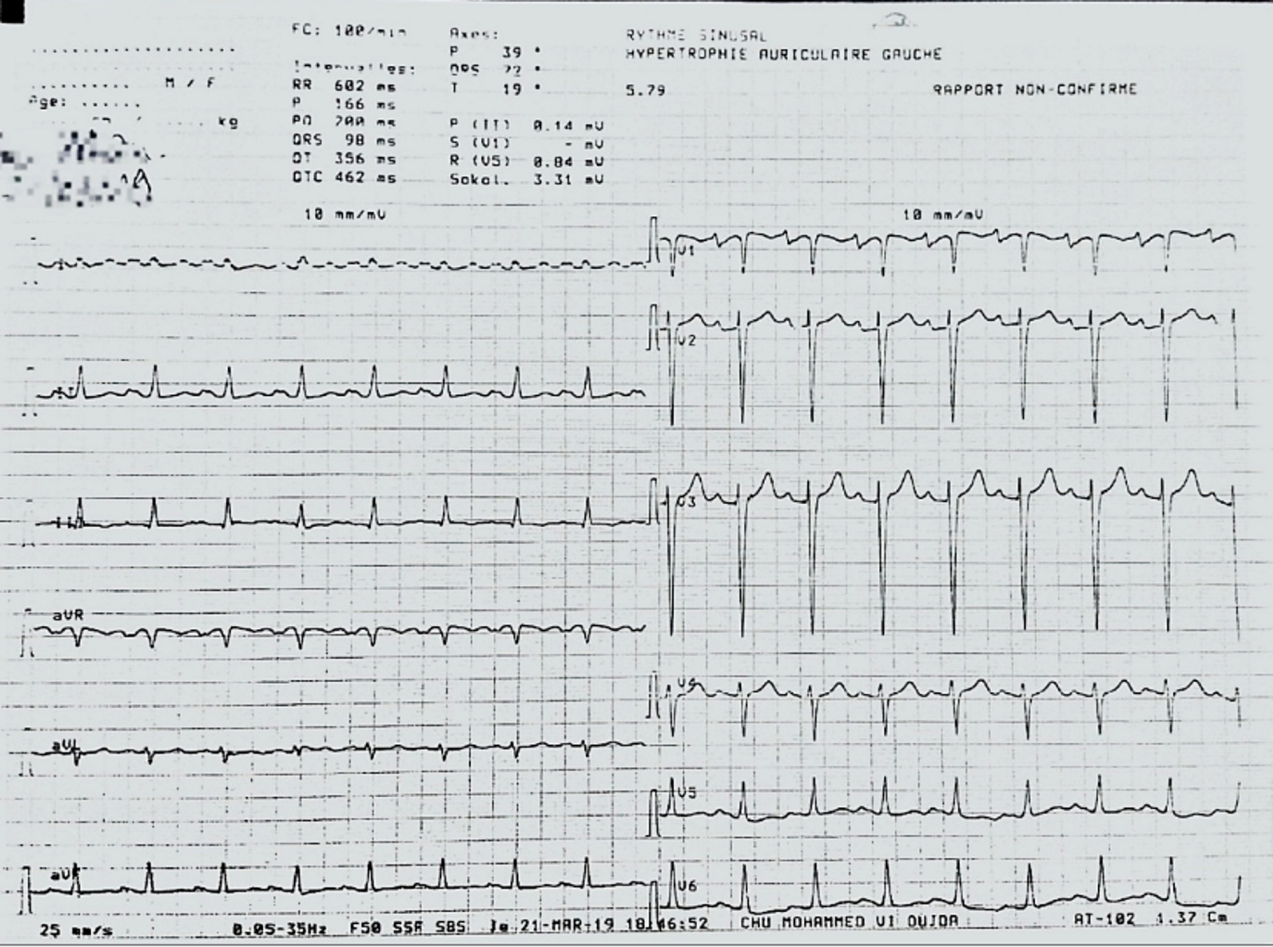
Electrocardiogram showing sinus tachycardia

We report the case of a 38-year-old female, multiparous G5P5, without any cardiovascular risk factors. The patient had a six-year history of dilated cardiomyopathy, probably of the peripartum, with severe left ventricular (LV) dysfunction [ejection fraction (EF) of 33%], discovered three months after her fourth childbirth. She was put under medical treatment with a favorable initial outcome. The patient was admitted to the cardiological intensive care unit for an acute global heart failure complicated by cardiogenic shock, one month after an emergency cesarean section at 34 weeks of gestation due to fetal distress. On examination, the patient was orthopnoeic with bilateral crackles on chest auscultation. She had lower limb edema with a positive Homan's sign. She had a pulse of 100/min with a low blood pressure of 80/60 mmHg. Chest X-ray showed cardiomegaly, pulmonary edema, and a right basilar focus of infection. The electrocardiogram revealed sinus tachycardia with paroxysmal atrial fibrillation (Figure 1).

**Figure 2:**
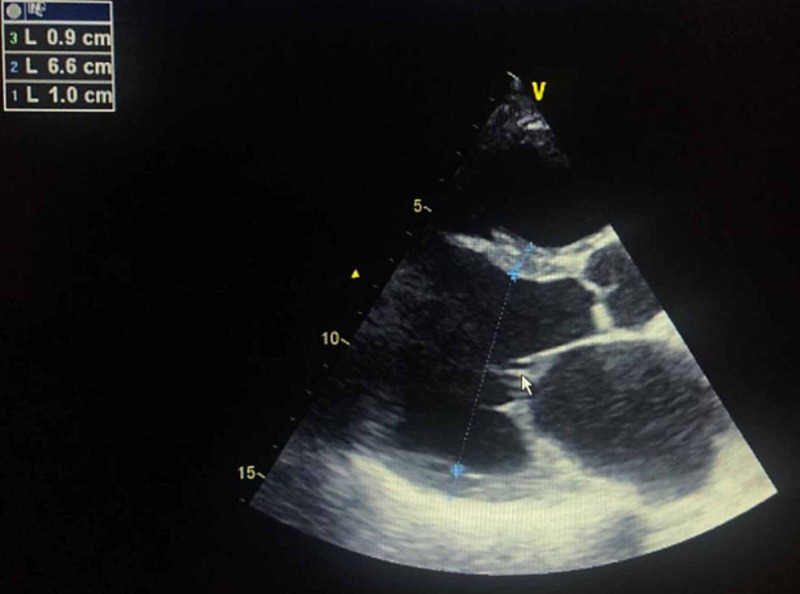
Transthoracic echocardiography showing left ventricular dilatation

Echocardiography showed biventricular dilated cardiomyopathy with an elevated filling pressure of LV. The end-diastolic dimension of the LV was 6.6 cm with global hypokinesia and severe systolic dysfunction (EF of 20%) along with grade II functional mitral regurgitation and severe pulmonary hypertension of 83 mmHg (Figure 2).

**Figure 3:**
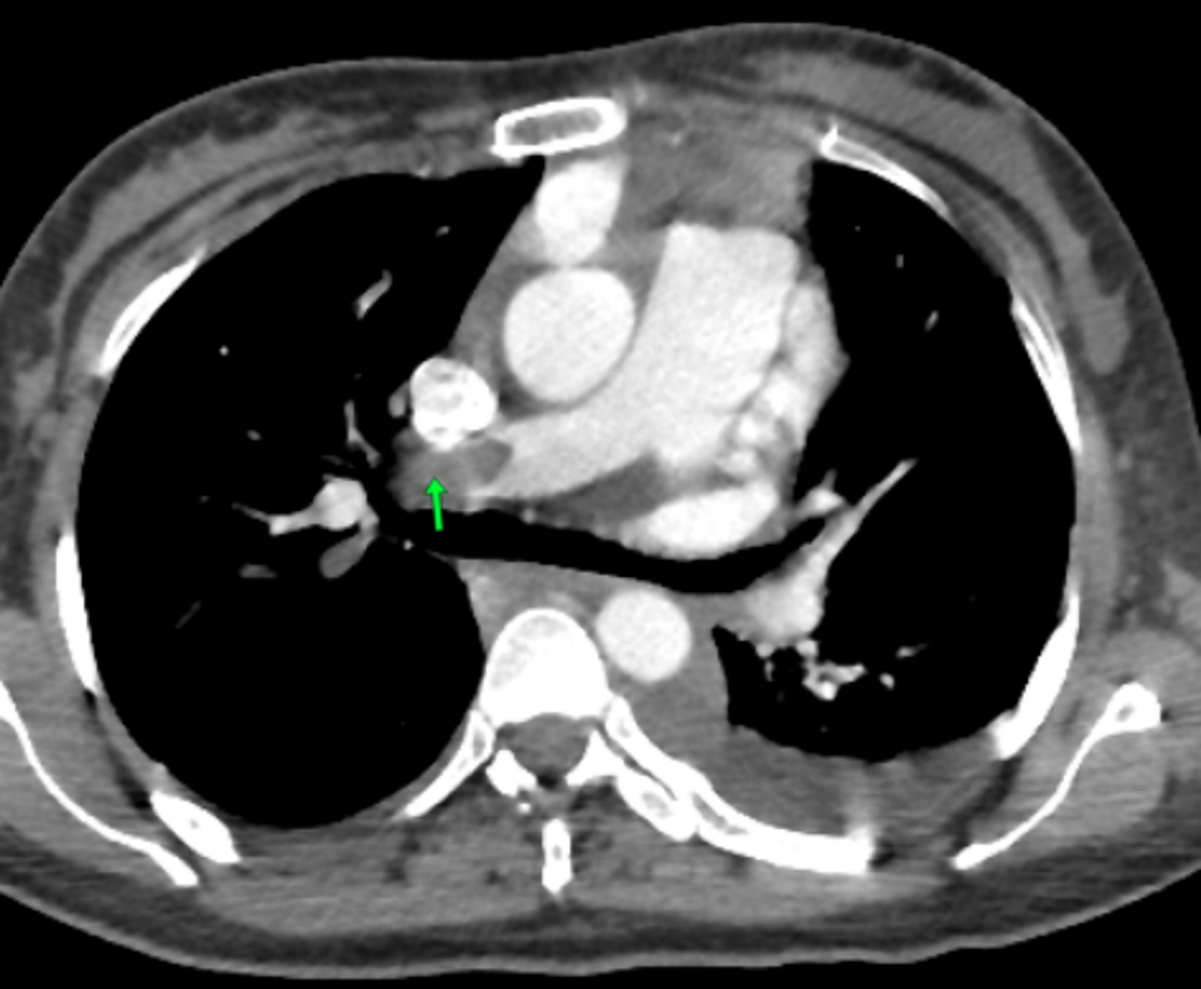
Chest CT angiogram shows embolic occlusion of the right pulmonary artery (arrow) CT: computed tomography

Given the clinical context, a thoracic CT angiography was performed, which revealed a massive pulmonary embolism of the right pulmonary artery extended to the right lobar and segmental branches of division at high intermediate risk with common right femoral deep vein thrombosis (Figure 3).

**Figure 4:**
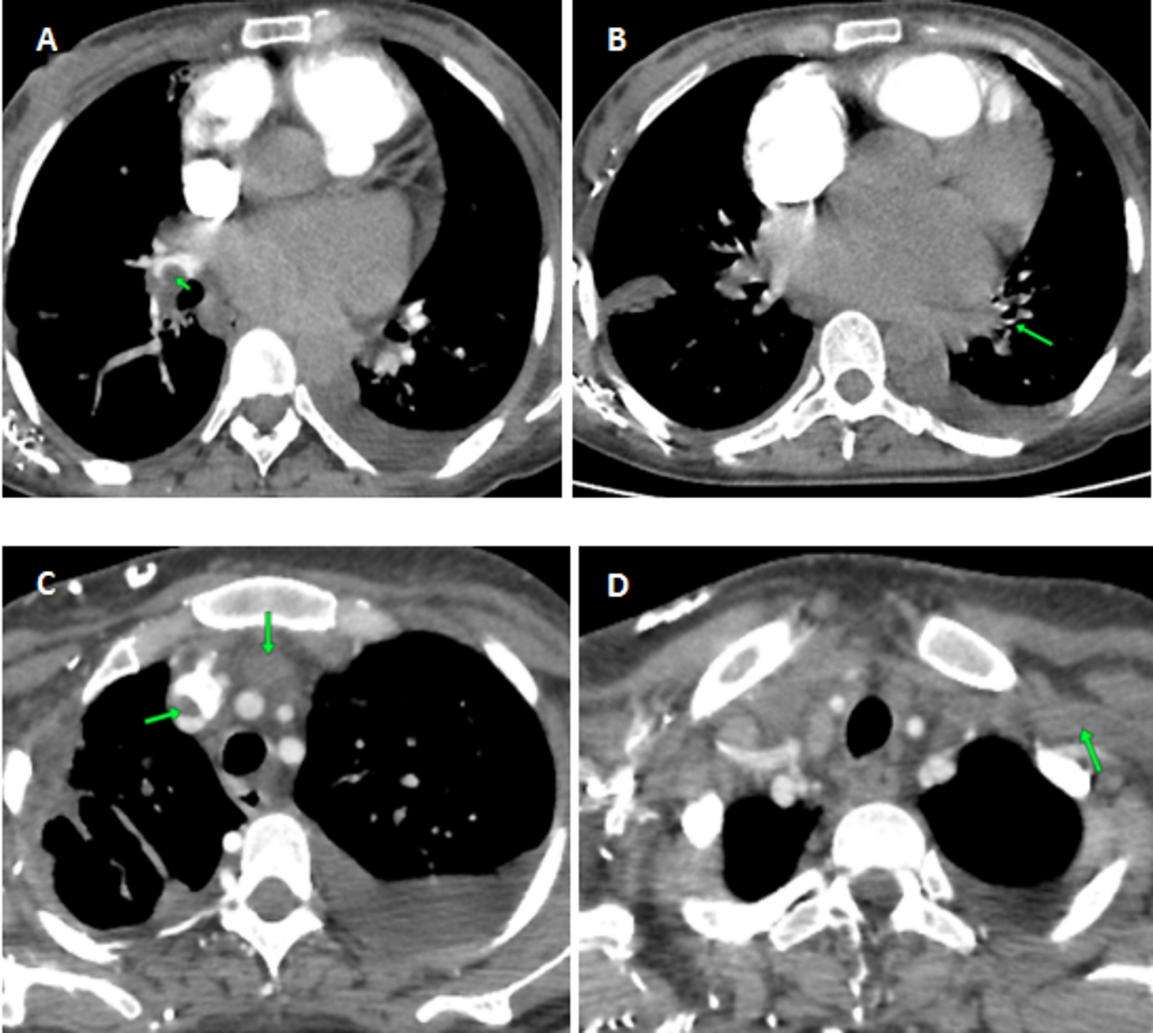
Chest CT angiogram during the second hospitalization A: right pulmonary embolism (arrow). B: pulmonary embolism of left segmental branches (arrow). C: deep vein thrombosis of the innominate venous trunk and the origin of the superior vena cava (arrows). D: deep vein thrombosis of the subclavian vein (arrow) CT: computed tomography

Laboratory investigations revealed elevated levels of brain natriuretic peptide at 8,276 ng/l and hypochromic anemia at 10 g/dl. C-reactive protein (CRP) levels were raised at 400 mg/l related to an infection of the foci of pulmonary infarction. Evaluation for secondary causes of dilated cardiomyopathy including thyroid hormone profile, viral serologies, vitamin, angiotensin-converting enzyme, and phosphocalcic dosages, searches for antinuclear antibodies, and the iron status returned normal, thereby allowing to retain the diagnosis of peripartum cardiomyopathy (PPCM). The evolution under medical treatment combining diuretics, angiotensin-converting enzyme inhibitors, beta-blockers, amiodarone, oral anticoagulation with acenocoumarol with all international normalized ratio (INR) values within the target range (2-3), and antibiotic therapy was marked by reappearance, one month later, of another episode of global heart failure with superior vena cava syndrome. Thoracic CT angiography revealed bilateral pulmonary emboli of the right pulmonary artery extended to the right lobar and segmental branches of division and of the left segmental branches associated with an extensive deep vein thrombosis of both jugular veins, sigmoid sinuses, subclavian veins, innominate venous trunks, and the origin of the superior vena cava with collateral venous circulation (Figure 4).

**Figure 5:**
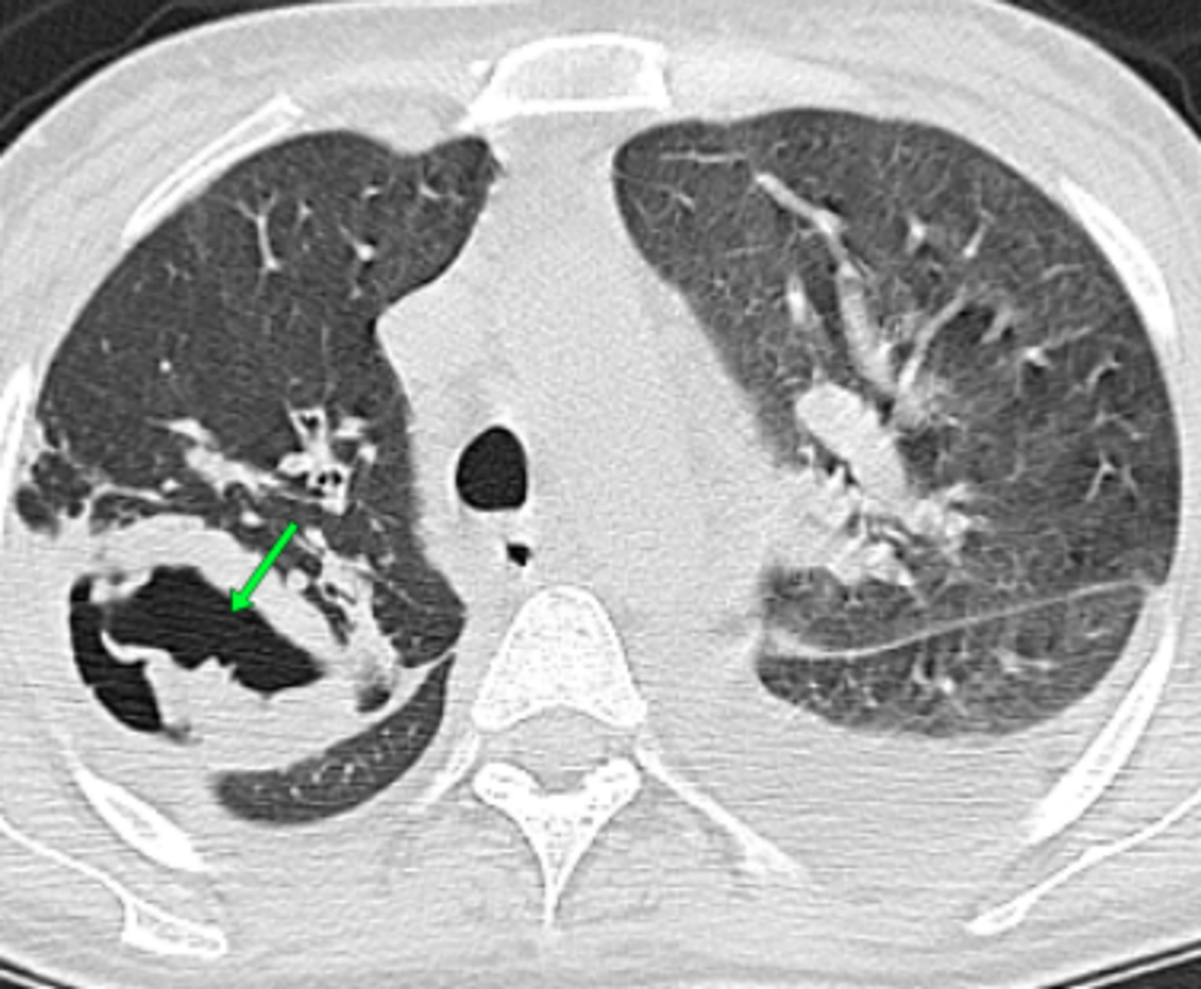
Chest CT scan showing a large excavated lesion caused by aspergillus in the right lung (arrow) CT: computed tomography

The patient was put on injectable diuretics and heparin of low molecular weight. Thrombophilia screen performed six weeks after the suspension of vitamin K antagonists (VKAs) revealed severe protein C deficiency with a rate of 37% (normal range: between 80-130%) and severe protein S deficiency with a rate of 40% (normal range: between 60-130%). The intra-hospital course was marked by an ominous refractory heart failure with pulmonary aspergillosis transplant and an increase in the inflammatory syndrome despite adapted antibiotic therapy, leading to patient death despite resuscitation measures (Figure 5).

## Discussion

PPCM is a rare dilated cardiomyopathy that accounts for less than 1% of heart diseases associated with pregnancy; its incidence varies between 1/3,000 to 1/15,000 live births and varies by geographical region [2]. It is defined as a congestive heart failure secondary to LV dysfunction that occurs in previously healthy women during the last month of pregnancy or during the five months after delivery with echocardiographic LV dysfunction (EF of <45%) and/or LV end-diastolic diameter of >2.7cm/m^2^ and without any other identifiable etiology; these were the criteria based on which we retained the diagnosis in our patient [3,4]. Several risk factors for this condition have been proposed, including maternal age of >30 years, black race, multiparity, twin pregnancies, obesity, hypertension and pre-eclampsia, prolonged tocolysis, and malnutrition; however, none of these risk factors would identify patients at high risk enough for PPCM to justify systematic screening [5].

The pathogenesis of PPCM remains unclear although several hypotheses have been proposed. An inflammatory theory has suggested that PPCM is triggered by myocarditis precipitated by disturbances of the inflammatory response during pregnancy, based on the findings of endomyocardial biopsies that displayed evidence of acute myocarditis and on the presence of serologic evidence of viral infection in the maternal blood, such as Coxsackie, parvovirus B19, Epstein-Barr virus (EBV), cytomegalovirus (CMV), and herpes virus [5]. However, an investigation on cardiomyopathies associated with pregnancy carried out on a cohort of 40 women who underwent cardiac MRI found lesions consistent with myocarditis in only one case [6]. Abnormal maternal immunological responses have been suggested resulting in the production of autoantibodies due to the passage of fetal cells into the maternal circulation or the production of anti-actin and myosin antibodies secondary to the degradation of uterine tropocollagen during delivery, which has a cross affinity with myocardial cells. A hormonal theory has proposed that the increase in intramyocardial oxidative stress in postpartum secondary to the sudden drop in estrogen levels activates cathepsin D (protease) that cleaves prolactin into a low molecular weight peptide (16KDa prolactin) with antiangiogenic and proapoptotic activity responsible for inadequate myocardial blood supply and contractile dysfunction of cardiomyocytes [2,7]. The presence of familial forms has also been reported, suggesting a genetic predisposition [7]. Other theories have been proposed incriminating nutritional disorders as well as mutations in the mitochondrial cytochrome b [5,7].

Patients typically present with symptoms of congestive heart failure either in the form of acute lung edema or gradually worsening dyspnea, which often leads to diagnostic delay. The electrocardiogram may be normal or show sinus tachycardia, atrial arrhythmia, atrial enlargement, or LV hypertrophy and often reveals non-specific ST segment or T wave abnormalities [5,7]. Chest X-ray usually shows cardiomegaly and signs of pulmonary edema. Echocardiography is the key cardiac imaging; it helps in confirming the diagnosis, rolling out the differential diagnoses, and also serves as a tool to ensure follow-up evaluation [5,7]. Cardiac MRI shows a late and no systematized enhancement after the injection of gadolinium, predominantly subepicardial [8], and whose intensity seems to correlate with a reserved prognosis in terms of LV function recovery, the occurrence of arrhythmias, and episodes of heart failure and mortality as reported in the largest series to date by Arora et al. [8]. Invasive hemodynamic investigations are indicated in severe presentations, and they allow for the simultaneous realization of coronary angiography in order to rule out a coronary artery disease when it is suspected and the performance of endomyocardial biopsies [5].

Several complications can be observed during PPCM, including heart failure comprising cardiogenic shock, cardiac arrhythmias, and thromboembolic complications, which are relatively frequent with an incidence in the United States ranging from 6.6% to 50% as per the prospective study by Avila et al. [9,10]. Thromboembolism is indicated by the combination of several conditions, including hypercoagulable state of pregnancy relating to hormonal changes that persist for several weeks after delivery, dilatation of cardiac chambers and endothelial inflammatory injuries as well as by venous stasis secondary to prolonged bed rest after cesarean section or instrumental deliveries [7]. In addition, the drop in cardiac output observed during hypokinetic cardiomyopathies including PPCM further promotes all types of thrombosis and embolism including deep vein thrombosis, pulmonary embolism, intracardiac thrombi, arterial embolism, and ischemic stroke [11,12]. Thrombophilia screening is also recommended despite the presence of these obvious thromboembolic risk factors, especially in young patients in whom thrombophilia is common. In the study conducted by Erbay et al. involving 60 patients with dilated cardiomyopathy, resistance to activated protein C was found in 12 of 22 patients (54%) with LV thrombus [13]. Paç FA and Cağdaş DN also concluded in their study that patients with dilated cardiomyopathy should be evaluated for other risk factors of thromboembolism and bleeding disorders and should be anticoagulated if any of these disorders is present [14].

Management of thromboembolic complications associated with PPCM is similar to that of other forms related to other etiologies [5]. However, our patient presented the particularity of thromboembolic recurrence (addition of other thrombotic sites) under well-conducted oral anticoagulation by acenocoumarol with all INR values within the target range (2-3), which is a rare situation observed in only 1-3% of cases and which may be explained by the physiopathological mechanisms mentioned above and also by protein C and protein S deficiency discovered in our patient, which has been widely described as a factor in the recurrence of thromboembolic disease [15,16,17]. In case of recurrence, it is recommended to consider switching to another VKA class: such as the switch to low-molecular-weight heparin (LMWH) as indicated in our patient or the combination of VKA and aspirin particularly in patients with antiphospholipid antibody syndrome (APS) [15]. New oral anticoagulants (NOACs) could represent a promising alternative as well [16]. In fact, three meta-analyses evaluating NOACs compared to VKAs in the treatment of venous thromboembolism disease have shown a similar efficiency in terms of thromboembolic events recurrences and a significant reduction in the risk of major bleeding with NOACs compared to VKAs [18]. For example, in the EINSTEIN-DVT study, the no inferiority of rivaroxaban was obtained with 2.1% recurrences under rivaroxaban versus 1.8% on enoxaparin-VKA (95% CI: 0.44-1.04; p: <0.001) [18]. In patients with thrombophilia, NOACs can have advantages and disadvantages according to Zuily S, who highlighted the role of NOACs in the preservation of protein C and protein S levels in the acute phase of venous thrombosis compared to VKAs [19]. Martinelli et al. have also demonstrated that rivaroxaban can be considered as a valid anticoagulation alternative in the treatment of recurrent thrombosis in patients with severe hereditary protein S deficiency and skin necrosis induced by warfarin [20].

The conventional treatment of heart failure is indicated in PPCM; it aims to decrease preload and afterload and improve myocardial contraction [5]. It is associated with bed rest, hydrosodic restriction, loop diuretics, nitrates, and cardioselective beta-blockers; angiotensin-converting enzyme inhibitors and angiotensin receptor blockers may be added after delivery. Administration of ivabradine or the association of sacubitril (neprilysin inhibitor) and valsartan are contraindicated during pregnancy when there is no data available during postpartum [7]. Intravenous vasoactive medications including dopamine, dobutamine, and levosimendan can be used in patients who do not respond to conventional treatments. When resistance is encountered, immunoglobulins and/or immunosuppressants may be used, and, more recently, bromocriptine (anti-prolactin) has proven its effectiveness in the treatment of patients with PPCM [5,7]. In cases of refractory cardiogenic shock, extracorporeal circulatory support is necessary as a bridge to recovery or before cardiac transplantation, which constitutes the last alternative in patients remaining symptomatic under maximal medical treatment, despite a greater risk of rejection compared to idiopathic dilated cardiomyopathies [5]. In a study carried out in the United States between 2004 and 2011, 2.6% of included women with PPCM had a cardiogenic shock; extracorporeal circulation assistance was necessary in 1.5% of cases, and 0.5% of women received a heart transplant [9]. In the Investigations of Pregnancy Associated Cardiomyopathy (IPAC) cohort that enrolled 100 patients, four women underwent mechanical circulatory assistance and one of them received a heart transplant. Unfortunately, our patient did not benefit from cardiac transplantation due to its unavailability and the rapidly fatal evolution of her condition. Because of the high frequency and severe impact of thromboembolism associated with PPCM, anticoagulant treatment is recommended according to the European Society of Cardiology (ESC) guidelines in patients with LVEF of <35% and in those who have received treatment with bromocriptine; the American Heart Association (AHA) guidelines advise considering anticoagulation in cases with LVEF of <30% while other experts have recommended anticoagulation in all women with PPCM until eight weeks after childbirth [4,7]. Ending the pregnancy or early delivery can sometimes be justified when the heart failure worsens despite optimal medical treatment and any subsequent pregnancy is contraindicated in women whose LV function remains impaired; however, in women who have recovered normal ventricular function, pregnancy remains possible but should be under strict supervision given the risk of recurrence [5,7]. The duration of medical treatment is not well defined and it is generally guided by ultrasound findings during follow-ups.

The prognosis of PPCM is often favorable with the recovery of the LV function reported in 50-80% of cases [7]. However, multiple factors of poor prognosis have been reported, including black race, older age, multiparity, and also persistent symptoms of heart failure for more than two weeks after delivery as well as the incidence of LV dilatation and a very low LV systolic function. The mortality rate related to PPCM is around 30% [7].

## Conclusions

Thromboembolic complications are common in patients with peripartum dilated cardiomyopathy and are an important factor affecting the prognosis of these patients, which justifies their systematic prevention by effective anticoagulation. We believe that patients with PPCM, and those presenting with thromboembolic events, should be evaluated for other thromboembolic risk factors including hemostatic disorders. In the event of thromboembolic recurrence, therapeutic management remains a real challenge, and further, large-scale randomized trials need to be conducted about the topic.
